# Assessment of Pharmacokinetic Drug Interaction of Asciminib with Atorvastatin in Healthy Participants

**DOI:** 10.1002/cpdd.1611

**Published:** 2025-10-22

**Authors:** Matthias Hoch, Wendy Weis, Felix Huth, Seshulatha Jamalapuram, Michelle Quinlan, Amarnath Bandaru, Suleyman Eralp Bellibas, Asmae Mirkou, Shruti Kapoor, Shefali Kakar

**Affiliations:** ^1^ Novartis Biomedical Research Basel Switzerland; ^2^ Novartis Pharmaceuticals East Hanover New Jersey USA; ^3^ Novartis Healthcare Pvt Ltd Hyderabad India; ^4^ Novartis Pharma AG Basel Switzerland

**Keywords:** asciminib, atorvastatin, CML, CYP3A4, drug‐drug interaction, OATP1B, pharmacokinetics

## Abstract

Asciminib is the first BCR::ABL1 inhibitor that Specifically Targets the ABL Myristoyl Pocket (STAMP) in patients with chronic myeloid leukemia. This phase 1, two‐treatment‐period, drug‐drug interaction study evaluated the effect of steady‐state asciminib on the pharmacokinetics of atorvastatin. A single dose of atorvastatin (20 mg) was administered on day 1 (period 1: days 1‐4). On days 5‐11 (period 2: days 5‐12), 80 mg asciminib was administered once daily, with a single dose of atorvastatin co‐administered on day 9. Pharmacokinetic sampling for atorvastatin was performed on days 1‐4 in period 1 and days 9‐12 in period 2. Twenty‐two healthy participants were enrolled. Twenty participants completed the study, and two discontinued due to adverse events (AEs). Asciminib increased the adjusted geometric mean (G_mean_) of maximum plasma concentration (C_max_), the area under the curve (AUC) from zero to the last quantifiable concentration (AUC_last_), and the AUC from zero to infinity (AUC_inf_) of atorvastatin by 24%, 16%, and 14%, respectively, and did not affect these parameters for its active metabolites, *o*‐hydroxy‐atorvastatin and *p*‐hydroxy‐atorvastatin. Plasma concentrations of coproporphyrin‐1 (CP‐1), an endogenous substrate of the atorvastatin transporter OATP1B, were not affected by asciminib. Thirteen participants reported at least one AE, all being grade 1/2, except for one grade 3 AE (increased alanine aminotransferase). No serious AEs were reported. In conclusion, concomitant administration of steady‐state asciminib and atorvastatin resulted in a small, clinically irrelevant increase in atorvastatin exposure and no change in CP‐1 concentrations. Both drugs were well tolerated. These data support co‐administration of asciminib and atorvastatin.

Chronic myeloid leukemia (CML) is a clonal myeloproliferative disorder characterized by uncontrolled proliferation of hematopoietic stem cells.^1^ The incidence of CML is 1 to 2 cases per 100,000 adult population.^2^ The vast majority of patients with CML have the Philadelphia (Ph) chromosome leading to the formation of the *BCR::ABL1* gene fusion, which encodes the constitutively active BCR::ABL1 tyrosine kinase providing a growth advantage to hematopoietic stem cells.^3^, ^4^ Tyrosine‐kinase inhibitors (TKIs) targeting BCR::ABL1 are currently a key therapeutic choice for patients with CML.^5^ Asciminib is the first BCR::ABL1 inhibitor that Specifically Targets the ABL Myristoyl Pocket (STAMP) resulting in the allosteric inhibition of ABL1.^6^ It is approved worldwide for the treatment of adult patients with Ph chromosome‐positive (Ph+) CML in chronic phase (CP) who have been previously treated with at least two TKIs, and in some countries, for the treatment of adult patients with the T315I BCR::ABL1 mutation.^7^, ^8^ Recommended doses of asciminib are 80 mg orally once daily (QD) or 40 mg twice daily (BID) in Ph+ CML in CP and 200 mg orally BID in Ph+ CML in CP with the T315I BCR::ABL1 mutation.^7^, ^8^ Asciminib has recently received approval for the treatment of newly diagnosed adult patients with Ph+ CML‐CP.^8^

Asciminib is rapidly absorbed, with plasma concentrations generally exhibiting a bi‐phasic decline after achieving maximum plasma concentration (C_max_). The median time to maximum observed plasma drug concentration (T_max_) of asciminib is 2‐3 h, regardless of the dose. Steady‐state levels for asciminib are achieved within 3 days of dosing, with an apparent terminal half‐life (T_1/2_) ranging between 7 and 15 h.^9^ For steady‐state asciminib, the geometric mean (G_mean_) of area under the curve (AUC) from zero to 24 h (AUC_0‐24_) was 15,112 ng × h/mL and C_max_ was 1781 ng/mL for the 80 mg QD regimen, while the AUC from zero to 12 h (AUC_0‐12_) was 5262 ng x h/mL and C_max_ was 793 ng/mL for the 40 mg BID regimen.^8^, ^9^

The median age of patients at the time of CML diagnosis typically ranges from 57 to 64 years across European countries and the USA,^10^, ^11^ and patients often present with cardiovascular risk factors or comorbidities.^12^, ^13^, ^14^ Observational studies have shown a direct relationship between low‐density lipoprotein (LDL) cholesterol levels and the occurrence of coronary heart disease.^15^ In addition, numerous studies have highlighted the significant role of LDL‐lowering medications, such as statins, in reducing the mortality and morbidity rates in patients with cardiovascular diseases.^15^ Atorvastatin is a statin commonly prescribed to reduce the risk of cardiovascular disease by lowering LDL‐cholesterol in hyperlipidemia.^16^ Over 85% of atorvastatin is metabolized by cytochrome P450 3A4 (CYP3A4), resulting in the formation of two active primary metabolites, *o*‐hydroxy‐atorvastatin and *p*‐hydroxy‐atorvastatin.^17^ Atorvastatin is transported by organic anion transporting polypeptide (OATP) 1B1 (uptake clearance of 52%),^18^ OATP1B3 (uptake clearance of 42%),^18^ P‐glycoprotein (P‐gp), and breast cancer resistance protein (BCRP).^19^, ^20^, ^21^ In the small intestine and the liver, P‐gp contributes to 43‐79% and 66% of the total efflux of atorvastatin, respectively, whereas BCRP contributes to 7.1‐8.8% and 4.5%, respectively.^19^, ^20^, ^21^

Plasma levels of atorvastatin increased with concomitant administration of transport inhibitors (e.g. single dose rifampin for OATP1B),^22^ and concomitant administration of atorvastatin with strong CYP3A4 inhibitors, including clarithromycin, human immunodeficiency virus protease inhibitor combinations, and itraconazole, is not recommended according to prescribing information.^23^ In clinical drug‐drug interaction (DDI) studies, asciminib was a weak inhibitor of CYP3A4,^9^, ^24^ whereas in vitro data have suggested that asciminib can inhibit the transporters OATP1B1, OATP1B3, P‐gp, and BCRP.^9^ These data suggest that asciminib may have the potential to increase atorvastatin exposure in the clinical setting. An increase in statin exposure could result in statin‐associated adverse events (AEs) such as myopathy and life‐threatening rhabdomyolysis.^9^, ^25^, ^26^ The risk of statin‐associated myopathy in a meta‐analysis of large, long‐term randomized‐controlled trials (RCTs) has been shown to be <0.1% relative to placebo, with the risk increasing when higher doses of statins or interacting drugs were administered.^25^ In some RCTs that investigated atorvastatin, a slightly higher rates of muscle symptoms were reported in the atorvastatin than placebo arms.^25^ The risk of rhabdomyolysis during statin treatment was shown to be around 0.01%.^25^ For atorvastatin, simvastatin, and pravastatin monotherapies, the rate of hospitalization due to rhabdomyolysis was 0.44 per 10,000 patient‐years (95% CI: 0.20‐0.84).^25^

Assessing transporter‐mediated DDIs using endogenous biomarkers to gauge a drug's potential to inhibit transporters has gained significant attention. Coproporphyrin (CP)‐1 and CP‐3 are organic anions and byproducts of heme biosynthesis that are absorbed by hepatocytes via the transporters OATP1B1 and OATP1B3.^27^, ^28^ Based on clinical evidence, CP‐1 and CP‐3 have been suggested as endogenous biomarkers of OATP1B activity.^27^, ^28^ Administration of a single dose of rifampicin, a potent OATP inhibitor, in healthy participants, led to an increase in C_max_ of CP‐1 and CP‐3 by 5.7‐ and 5.4‐fold, respectively.^28^ The utility of CP‐1 as an endogenous biomarker for OATP1B‐mediated DDIs was evaluated using a nonlinear mixed‐effect modeling approach, which demonstrated sensitivity of CP‐1 to identify moderate and weak inhibitors of OATP1B in a well‐powered clinical study.^29^ Therefore, CP‐1 is now an established biomarker for OATP1B inhibition.

The results of a phase I study that aimed to evaluate the effect of steady‐state asciminib on the plasma PK profile of atorvastatin, its metabolites, and CP‐1 in healthy adult participants are presented in this article.

## Methods

### Study Design and Treatment Administration

**Figure 1:**
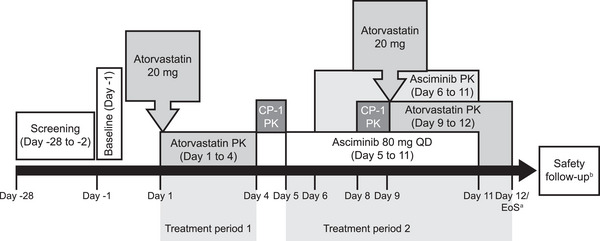
Study design. ^a^EoS defined as date of last scheduled procedure. ^b^A post‐study safety contact (e.g. follow‐up telephone call, email) occurred approximately 30 days after the last administration of study treatment. CP‐1, coproporphyrin‐1; EoS, end of study; PK, pharmacokinetics; QD, once daily.

This was a phase I, open‐label, single‐sequence DDI study. The study consisted of a screening period, two treatment periods, and a safety follow‐up of 30 days after the last dose of asciminib (Figure 1).

During treatment period 1 (days 1‐4), a single oral dose of atorvastatin 20 mg was administered to participants on day 1 after an overnight fast of at least 10 h. Participants were required to fast for 4 h after atorvastatin dosing. There was a 4‐day washout period between the first dose of atorvastatin in period 1 and the first dose of asciminib in period 2. During treatment period 2 (days 5‐12), asciminib 80 mg QD was administered to participants from day 5 to day 11, with a single oral dose of atorvastatin (20 mg) co‐administered with asciminib on day 9. This timing was determined based on asciminib's PK profile, which shows that steady‐state is reached within 3 days of dosing.^9^ Participants were required to fast for at least 2 h before and for 1 h after asciminib dosing, except on day 9 when asciminib and atorvastatin were co‐administered, and participants fasted for at least 10 h prior to and 4 h after dosing. All doses of asciminib were administered approximately 24 h apart (±1 h). Asciminib and atorvastatin tablets were taken with approximately 240 mL of water. No fluid intake was allowed 1 h pre‐ and 1 h post‐dosing.

No medication other than study treatment was allowed from the date the participant signed the informed consent until all end of study evaluations were performed, except for protocol‐allowed concomitant medications and/or medication which may have been required to treat AEs. The consumption of foods or beverages known to inhibit or induce CYP3A4 or uridine 5′‐diphospho‐glucuronosyltransferase (fruits [including grapefruit, Seville oranges, pomelos, pomegranates, cranberries and star fruits] and cruciferous vegetables [including kale, broccoli, cabbage, cauliflower, watercress, collard greens, kohlrabi, Brussels sprouts, and mustard greens]) were to be avoided from 14 days prior to dosing until the end of study.

### Participants

Eligible participants were healthy male or female (sterile or post‐menopausal) adults (aged 18‐55 years). Key eligibility criteria were a body mass index (BMI) between 18.5 and 29.9 kg/m^2^, body weight of at least 50 kg, body temperature between 35.0 and 37.5°C, systolic blood pressure from 90 to 139 mmHg, diastolic blood pressure from 50 to 89 mmHg, and pulse rate from 45 to 100 beats per minute (bpm) at screening, and good health as determined by no clinically significant findings from medical history, physical examination, vital signs, electrocardiogram (ECG), and routine laboratory tests.

Key exclusion criteria were a history of any clinically significant medical conditions, having been treated with investigational drugs within 30 days (or 5 half‐lives, whichever is longer) prior to study dosing, contraindications, hypersensitivity or any previous adverse reaction to asciminib, or a history of any AE potentially related to previous exposure to a statin or any other lipid‐lowering drug.

### Objectives and Endpoints

The primary objective was to evaluate the effect of asciminib at steady state on the single‐dose PK of atorvastatin by measuring primary PK parameters for atorvastatin, including C_max_, the AUC from zero to the last quantifiable concentration (AUC_last_), and the AUC from zero to infinity (AUC_inf_), and secondary PK parameters for atorvastatin, such as T_max_ and T_1/2_. Secondary objectives included: (1) assessment of the effect of asciminib at steady state on the single‐dose PK of atorvastatin metabolites by measuring C_max_, AUC_last_, AUC_inf_, T_max_, and T_1/2_ for *o*‐hydroxy‐atorvastatin and *p*‐hydroxy‐atorvastatin; (2) evaluation of the safety and tolerability of multiple doses of asciminib given alone or concomitantly with atorvastatin based on the incidence of AEs and serious AEs (coded using Medical Dictionary for Regulatory Activities version 26.1 and Common Terminology Criteria for AEs version 5), changes in hematology and blood chemistry values, vital signs, and ECG; (3) assessment of the effect of asciminib on CP‐1 by measuring the plasma concentration over the last 24‐h dosing interval and deriving the AUC_0‐24_; and (4) identification of the asciminib plasma trough drug concentration at the end of the dosing interval at steady state (C_trough_).

### PK Sampling and Assessments

On days 1 and 9, blood samples were collected before dosing and at 0.5, 1, 1.5, 2, 3, 4, 5, 6, 8, 10, 12, 24, 36, 48, and 72 h after dosing to characterize the plasma PK of atorvastatin and its metabolites. Blood samples were collected before morning asciminib dosing on days 6 to 11 to characterize ascimininb C_trough_. Plasma was obtained for CP‐1 analysis on days 4 and 8 prior to asciminib dosing to attain baseline values (0 h) and at 0.5, 1, 1.5, 2, 3, 4, 5, 6, 8, 10, 12, and 24 h after dosing. All plasma samples were stored frozen at −60°C until analysis. Plasma concentrations of atorvastatin, *o*‐hydroxy‐atorvastatin, *p*‐hydroxy‐atorvastatin, asciminib, and CP‐1 were determined using validated liquid chromatography‐tandem mass spectrometry (LC‐MS/MS) assays, with a lower limit of quantification (LLOQ) of 0.1 ng/mL for atorvastatin and *o*‐hydroxy‐atorvastatin, 0.05 ng/mL for *p*‐hydroxy‐atorvastatin, 1.0 ng/mL for asciminib, and 50 pg/mL for CP‐1. PK parameters were derived from individual plasma concentration‐time profiles by non‐compartmental methods using Phoenix WinNonlin software version 8.3.

### Statistical Analysis

The safety set, which included all participants who received at least one dose of any study treatment (atorvastatin or asciminib), was used for safety evaluations. The PK analysis set for atorvastatin included all participants who provided an evaluable atorvastatin PK profile for at least one period for the analyte of interest (atorvastatin, *o*‐hydroxy‐atorvastatin, *p*‐hydroxy‐atorvastatin, or CP‐1). The PK analysis set for CP‐1 included all participants who provided an evaluable CP‐1 PK profile for at least one period. The PK analysis set for asciminib included all participants who provided at least one evaluable asciminib C_trough_ value in period 2.

Descriptive statistics were used for all PK parameters for atorvastatin, *o*‐hydroxy‐atorvastatin, *p*‐hydroxy‐atorvastatin, CP‐1, and asciminib C_trough_. A linear mixed‐effect model was fitted to the log‐transformed PK parameters (AUC_last_, AUC_inf_, and C_max_) to assess the effect of asciminib at steady state on the PK of a single dose of atorvastatin. The model included treatment as a fixed factor and participant as a random factor to obtain adjusted G_mean_ estimates. The difference between the means of the test (atorvastatin plus asciminib) and reference (atorvastatin) were calculated and back‐transformed to obtain G_mean_ ratios, and 90% CIs. Plasma concentrations below the LLOQ were set to zero, and treated as missing in calculations of G_mean_ and geometric coefficients of variation percentage (GCV%). All statistical analyses were performed using Statistical Analysis System (SAS) version 9.4.

### Sample Size

Based on previously reported data, the maximum inter‐participant coefficient of variation percentage (CV%) of AUC_last_, AUC_inf_, and C_max_ from atorvastatin was 68.6%.^30^ A conservative approach was followed by using an intra‐participant CV% of 41% (which equates to 60% of the inter‐participant CV%). Using an intra‐participant CV% of 41% and a sample size of 18 participants, the precision or halfwidth of the 90% CIs for test‐reference comparison on the logarithmic scale extended 0.229 from the observed difference in means. This calculation was based on a t‐test with two‐sided alpha level of 0.10 and 17 degrees of freedom, using the model with treatment as a fixed factor and participant as a random factor. Assuming a potential dropout rate of 20%, 22 participants were enrolled to ensure at least 18 evaluable participants satisfied the PK analysis set criteria.

### Ethics

The study was conducted according to the International Council for Harmonisation E6 Guideline for Good Clinical Practice in accordance with the principles of the Declaration of Helsinki, as well as local laws and regulations. All participants provided written informed consent before any study procedures took place. The study protocol and all amendments were reviewed by the independent ethics committee and/or institutional review board for each study center.

## Results

### Participant Disposition and Baseline Characteristics

All 22 participants enrolled in the study received a single dose of atorvastatin in period 1. In period 2, 20 participants received one dose of atorvastatin and all seven doses of asciminib, and 1 participant received only three doses of asciminib. Of the 22 participants, 20 completed the study and 2 discontinued early due to AEs.

The median age at baseline was 35.0 years (range: 22‐53 years). Most participants were male (19; 86.4%) and White (20; 90.9%), and all participants were of Hispanic or Latino ethnicity (Table 1). The median BMI (range) and weight (range) were within the protocol prespecified inclusion criteria (Table 1).

**Table 1:** Demographic and Baseline Characteristics

Demographic and baseline characteristics	Participants N = 22
Age, median (range), years	35.0 (22‐53)
Female, n (%)	3 (13.6)
Male, n (%)	19 (86.4)
Race, n (%)	
Black or African American	1 (4.5)
White	20 (90.9)
White, Black or African American	1 (4.5)
Ethnicity, n (%)	
Hispanic or Latino	22 (100.0)
Weight,^a^ median (range), kg	75.6 (57.2‐103.1)
Height,^a^ median (range), cm	169.5 (158‐191)
BMI,^a^ median (range), kg/m^2^	26.7 (22.9‐29.8)

^a^Descriptive statistics for weight, height, and BMI are calculated using screening measurements.

BMI, body mass index.

All prior and concomitant medications taken during the study were authorized per protocol and had no impact on interpretation of the study data. One participant received amoxicillin trihydrate (875 mg)‐clavulanate potassium (125 mg) per investigator decision for an AE of asymptomatic urinary tract infection during the study. The medication did not have an impact on PK data, since it was administered on day 12 after completion of PK sampling.

### DDI of Asciminib With Atorvastatin

**Figure 2:**
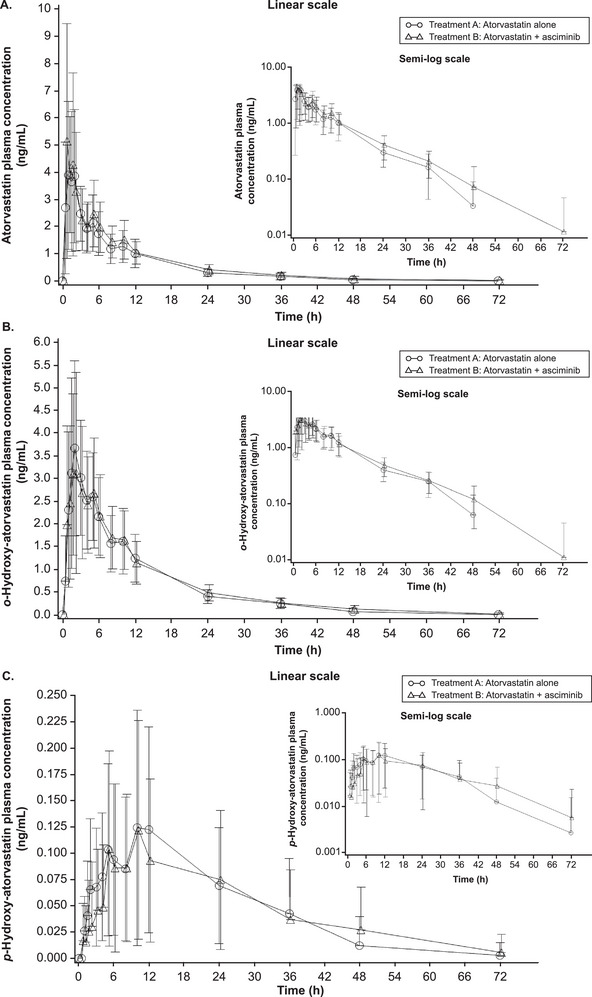
Arithmetic mean (standard deviation) plasma concentration‐time profiles of atorvastatin (A), *o*‐hydroxy‐atorvastatin (B), and *p*‐hydroxy‐atorvastatin (C) when atorvastatin was administered alone or with asciminib at steady state. Linear views are shown in the main panel, with semi‐logarithmic views in the top right‐hand corner. h, hours.

Following administration of atorvastatin alone or with asciminib, mean C_max_ of atorvastatin was observed at 1 and 0.5 h post‐dose, respectively, with atorvastatin plus asciminib having a higher peak than atorvastatin alone (Figure 2A). After reaching mean C_max_, atorvastatin concentrations decreased similarly for both treatments. Primary and secondary PK parameters are summarized in Table 2. Co‐administration of atorvastatin with asciminib slightly increased plasma atorvastatin mean C_max_, AUC_last_, and AUC_inf_ compared with these parameters for atorvastatin alone. The median T_max_ was similar for both treatments at approximately 1 h. G_mean_ T_1/2_ was similar for atorvastatin plus asciminib compared with atorvastatin alone (Table 2). Following atorvastatin alone and atorvastatin plus asciminib treatment, mean C_max_ of *o*‐hydroxy‐atorvastatin was observed at 2 h for both treatment groups, with a lower peak for atorvastatin plus asciminib than atorvastatin alone (Figure 2B). After reaching mean C_max_, *o*‐hydroxy‐atorvastatin mean C_max_ declined similarly for both treatments. Mean plasma *o*‐hydroxy‐atorvastatin C_max_, AUC_last_, and AUC_inf_ were comparable between treatments (Table 2). Median T_max_ for *o*‐hydroxy‐atorvastatin was earlier for atorvastatin plus asciminib (1.5 h) compared with atorvastatin alone (2 h), and the mean T_1/2_ was longer for atorvastatin plus asciminib (12.2 h) than atorvastatin alone (10.0 h). Mean C_max_ of *p*‐hydroxy‐atorvastatin was observed at 10 h post‐dose and declined similarly for both treatments (Figure 2C). Mean plasma *p*‐hydroxy‐atorvastatin C_max_, AUC_last_, and AUC_inf_ were comparable between treatments (Table 2). Median T_max_ was similar between treatments at approximately 10 h (Table 2).

**Table 2:** PK Parameters of Atorvastatin, *o*‐Hydroxy‐atorvastatin, and *p*‐Hydroxy‐atorvastatin Administered Alone or With Asciminib at Steady State

	Atorvastatin		*o*‐Hydroxy‐atorvastatin		*p*‐Hydroxy‐atorvastatin	
PK parameter	Atorvastatin	Atorvastatin + asciminib	Atorvastatin	Atorvastatin + asciminib	Atorvastatin	Atorvastatin + asciminib
C_max_ (ng/mL)						
*n*	22	20	22	20	17	15
Mean (SD)	5.84 (2.91)	7.51 (3.88)	4.14 (1.98)	4.27 (2.11)	0.189 (0.112)	0.171 (0.0880)
G_mean_ (GCV%)	5.29 (45.7)	6.45 (66.2)	3.66 (56.9)	3.98 (45.4)	0.17 (53.8)	0.16 (48.0)
Median (min, max)	4.66 (2.40, 12.9)	7.71 (1.86, 15.0)	4.04 (1.42, 8.11)	3.88 (1.56, 11.3)	0.144 (0.0793, 0.468)	0.145 (0.0699, 0.425)
AUC_last_ (ng x h/mL)						
n	22	20	22	20	17	15
Mean (SD)	34.1 (15.5)	39.6 (15.8)	39.8 (15.0)	41.7 (15.4)	3.73 (2.75)	3.83 (3.28)
G_mean_ (GCV%)	31.5 (40.1)	36.8 (40.7)	37.0 (42.2)	39.2 (36.8)	3.1 (69.8)	2.9 (87.7)
Median (min, max)	30.6 (18.0, 80.1)	38.8 (18.7, 80.0)	41.6 (15.2, 75.8)	40.1 (21.4, 79.2)	2.56 (0.964, 12.5)	2.99 (0.717, 13.6)
AUC_inf_ (ng x h/mL)						
n	22	20	21	20	4	6
Mean (SD)	36.3 (15.4)	42.0 (16.4)	43.4 (14.3)	44.1 (15.5)	8.16 (4.48)	7.67 (4.91)
G_mean_ (GCV%)	33.9 (38.0)	39.2 (39.6)	41.2 (34.7)	41.7 (34.9)	7.4 (53.2)	6.2 (95.2)
Median (min, max)	34.4 (19.2, 81.6)	40.6 (20.4, 82.6)	44.1 (23.1, 77.7)	42.8 (23.6, 81.7)	6.60 (4.86, 14.6)	6.63 (1.50, 15.7)
T_max_ (h)						
n	22	20	22	20	17	15
Median (min, max)	1.01 (0.50, 3.00)	1.00 (0.50, 3.99)	2.00 (0.50, 5.00)	1.50 (0.50, 6.00)	10.00 (1.50, 12.01)	10.00 (1.00, 12.00)
T_1/2_						
n	22	20	21	20	4	6
Mean (SD)	9.96 (4.16)	11.1 (3.91)	10.0 (2.95)	12.2 (3.43)	31.1 (7.50)	25.3 (10.9)
G_mean_ (GCV%)	9.15 (45.3)	10.5 (34.0)	9.64 (28.0)	11.7 (28.4)	30.4 (24.6)	22.8 (58.5)
Median (min, max)	9.04 (3.51, 19.7)	9.87 (5.25, 20.1)	9.67 (5.12, 19.9)	12.2 (7.33, 21.2)	30.4 (24.3, 39.3)	26.0 (8.45, 40.9)

AUC, area under the curve; AUC_inf_, AUC from zero to infinity; AUC_last_, AUC from zero to the last quantifiable concentration; C_max_, maximum concentration of drug in plasma; GCV, geometric coefficient of variation; G_mean_, geometric mean; h, hours; n, number of observations used for analysis; PK, pharmacokinetic; T_1/2_, terminal half‐life; T_max_, time to reach maximum concentration of drug in plasma.

In a statistical comparison of the PK parameters for atorvastatin plus asciminib versus atorvastatin alone, asciminib increased the adjusted G_mean_ of C_max_, AUC_last_, and AUC_inf_ for atorvastatin by 24%, 16%, and 14% (Table 3). The estimated plasma *o*‐hydroxy‐atorvastatin adjusted G_mean_, C_max_, AUC_last_, and AUC_inf_ were comparable between treatments (Table 3). The estimated plasma *p*‐hydroxy‐atorvastatin adjusted G_mean_, C_max_, and AUC_last_ were similar between treatments (Table 3).

**Table 3:** Statistical Comparison of Primary PK Parameters of Atorvastatin Administered Alone or With Asciminib at Steady State

					Treatment comparison	
PK parameter	Treatment	n	Adjusted G_mean_ ^a^	Comparison	G_mean_ ratio^a^	90% CI^a^
**Atorvastatin**						
C_max_ (ng/mL)	Atorvastatin	22	5.29	Atorvastatin + asciminib/atorvastatin	1.24	1.00‐1.53
	Atorvastatin + asciminib	20	6.54			
AUC_last_ (ng x h/mL)	Atorvastatin	22	31.5	Atorvastatin + asciminib/atorvastatin	1.16	1.06‐1.27
	Atorvastatin + asciminib	20	36.5			
AUC_inf_ (ng x h/mL)	Atorvastatin	22	33.9	Atorvastatin + asciminib/atorvastatin	1.14	1.06‐1.24
	Atorvastatin + asciminib	20	38.8			
***o*‐Hydroxy‐atorvastatin**						
C_max_ (ng/mL)	Atorvastatin	22	3.66	Atorvastatin + asciminib/atorvastatin	1.08	0.87‐1.33
	Atorvastatin + asciminib	20	3.95			
AUC_last_ (ng x h/mL)	Atorvastatin	22	37.0	Atorvastatin + asciminib/atorvastatin	1.07	0.97‐1.17
	Atorvastatin + asciminib	20	39.4			
AUC_inf_ (ng x h/mL)	Atorvastatin	21	40.7	Atorvastatin + asciminib/atorvastatin	1.02	0.95‐1.11
	Atorvastatin + asciminib	20	41.7			
***p*‐Hydroxy‐atorvastatin**						
C_max_ (ng/mL)	Atorvastatin	17	0.160	Atorvastatin + asciminib/atorvastatin	0.98	0.83‐1.16
	Atorvastatin + asciminib	15	0.156			
AUC_last_ (ng x h/mL)	Atorvastatin	17	2.96	Atorvastatin + asciminib/atorvastatin	0.98	0.74‐1.28
	Atorvastatin + asciminib	15	2.89			
AUC_inf_ (ng x h/mL)	Atorvastatin	4	8.04	Atorvastatin + asciminib/atorvastatin	0.74	0.33‐1.63
	Atorvastatin + asciminib	6	5.92			

^a^The linear mixed‐effects model included treatment as fixed effect and participant as a random effect. Results were back‐transformed to obtain the adjusted G_mean_, G_mean_ ratio, and 90% CI.

AUC_inf_, area under the plasma concentration‐time curve from zero to infinity; AUC_last_, area under the plasma concentration‐time curve from zero to the last quantifiable concentration; CI, confidence interval; C_max_, maximum plasma concentration; G_mean_, geometric mean; n, number of observations used for analysis; PK, pharmacokinetic.

### CP‐1 Plasma Concentrations

**Figure 3:**
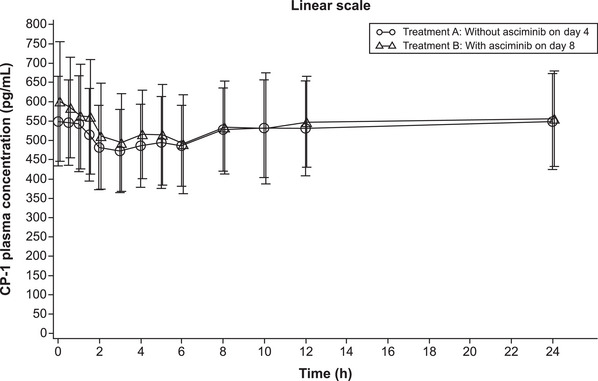
Arithmetic mean (standard deviation) CP‐1 plasma concentration‐time profile when atorvastatin was administered alone or with asciminib at steady state. CP‐1, coproporphyrin‐1; h, hours.

CP‐1 plasma concentrations remained constant throughout the sampling interval with minimal variation in both the absence and presence of asciminib over the complete sampling time period (i.e. 24 h) (Figure 3). The G_mean_ (GCV%) AUC_0‐24_ for CP‐1 when atorvastatin was administered without asciminib on day 4 was 12,300 pg x h/mL (21.8%) and with asciminib on day 8 was 12,600 pg x h/mL (23.6%). The adjusted G_mean_ AUC_0‐24_ for CP‐1 was comparable between treatments (Table 4).

**Table 4:** Statistical Comparison of CP‐1 Plasma AUC_0‐24_ When Atorvastatin was Administered with Steady‐state Asciminib Versus Without Asciminib

					Treatment comparison	
PK parameter	Treatment	n	Adjusted G_mean_ ^a^	Comparison	G_mean_ ratio^a^	90% CI^a^
AUC_0‐24_ (pg x h/mL)	Atorvastatin	21	12,300	Atorvastatin + asciminib/atorvastatin	1.02	0.98‐1.05
	Atorvastatin + asciminib	20	12,500			

^a^The linear mixed‐effects model included treatment as a fixed effect and participant as a random effect. Results were back‐transformed to obtain the adjusted G_mean_, G_mean_ ratio, and 90% CI. AUC_0‐24_, area under the curve from 0 to 24 h; CI, confidence interval; CP‐1, coproporphyrin‐1; G_mean_, geometric mean; n, number of observations used for analysis.

### PK Analysis of Asciminib

Steady state of asciminib (80 mg QD) was attained after 3 days of dosing (i.e. on day 8). Mean (standard deviation) C_trough_ of asciminib were 100 (28.3), 126 (33.5), 161 (62.2), 153 (62.2), 231 (71.1), and 173 (60.7) ng/mL on days 6, 7, 8, 9, 10, and 11, respectively (Figure S1).

### Safety

Overall, 13 of the 22 participants enrolled experienced at least one AE during the study (Table 5). The most common AE system organ class was skin and subcutaneous tissue disorders with 7 (31.8%) of the 22 participants reporting AEs, followed by investigations (4 [18.2%] participants), nervous system disorders (3 [13.6%] participants), and gastrointestinal disorders and respiratory, thoracic and mediastinal disorders (2 [9.1%] participants each). Overall, one grade 3 AE was reported (increased alanine aminotransferase [ALT]), and the remaining AEs were all grade 1 or 2. The most frequently reported AE was grade 1 pruritus (5 [22.7%] participants). Increased heart rate and rash were each reported in 3 (13.6%) participants. Headache and skin burning sensation were each reported in 2 (9.1%) participants. All remaining AEs were reported by 1 (4.5%) participant each. One participant reported laboratory‐related AEs of increased ALT, increased aspartate aminotransferase (AST), and increased gamma‐glutamyl transferase (GGT). Three participants had vital sign‐related AEs of increased heart rate and increased blood pressure.

**Table 5:** Summary of Treatment‐emergent Adverse Events by Preferred Term

Preferred term^a^	Participants N = 22
Participants with at least one event, n (%)	13 (59.1)
Pruritus	5 (22.7)
Heart rate increased	3 (13.6)
Rash	3 (13.6)
Headache	2 (9.1)
Skin burning sensation	2 (9.1)
Acne	1 (4.5)
ALT increased	1 (4.5)
AST increased	1 (4.5)
Blood pressure increased	1 (4.5)
Constipation	1 (4.5)
Dry skin	1 (4.5)
Dyspnea	1 (4.5)
GGT increased	1 (4.5)
Hyperesthesia	1 (4.5)
Oropharyngeal pain	1 (4.5)
Palpitations	1 (4.5)
Tongue discomfort	1 (4.5)
Urinary tract infection	1 (4.5)
Vessel puncture site reaction	1 (4.5)

^a^Percentages are based on the number of participants dosed. Adverse events were classified according to MedDRA version 26.1. A participant with multiple events was counted only once within a category. The same participant may appear in different categories.

ALT, alanine aminotransferase; AST, aspartate aminotransferase; GGT, gamma‐glutamyl transferase; MedDRA, Medical Dictionary for Regulatory Activities.

AEs leading to treatment discontinuation were reported in 2 participants. One participant who received atorvastatin 20 mg on day 1 of period 1 and only the first three doses of asciminib 80 mg QD in period 2 discontinued treatment due to increased ALT, increased AST, and increased GGT. The other participant received atorvastatin 20 mg on day 1 of period 1 and discontinued due to increased heart rate.

Three out of 21 (14.3%) participants reported a QT interval increase from >30 ms at baseline to ≤60 ms post‐baseline, 3 out of 22 (13.6%) participants reported a new PR interval >200 ms, 2 out of 22 (9.1%) participants reported a new QRS >110 ms, 1 out of 22 (4.5%) participants reported a QRS increase by >25% from baseline and QRS >110 ms, and 1 out of 21 (4.8%) participants reported an increase in heart rate from baseline by >25% and heart rate >100 bpm. No participants exhibited a QT corrected for heart rate using Fridericia's correction (QTcF) interval >450 ms or a QTcF increase from baseline by >30 ms. None of these ECG values were considered clinically significant nor were they reported as AEs by the investigator.

No serious AEs were reported in the study and no deaths occurred during the study.

## Discussion

This DDI study was conducted in healthy participants to assess the effect of asciminib at steady state on the PK parameters of a single dose of atorvastatin. At the time of CML diagnosis, patients often present with cardiovascular risk factors, and statins are frequently prescribed to lower the risk of cardiovascular diseases.^12^, ^13^, ^14^, ^31^ The results of this study provide evidence affirming that there is no relevant impact of asciminib on the PK of atorvastatin, thus supporting the combined use of asciminib with atorvastatin and other lipid‐lowering agents that utilize comparable metabolic and transport pathways (i.e. OATP1B).

Asciminib exhibited a weak DDI effect on atorvastatin by increasing the C_max_ of atorvastatin by 24% and overall exposure (AUC_last_ and AUC_inf_) by 14%‐16%, which is considered to be clinically irrelevant.^32^ The effect of asciminib on the production of *o*‐hydroxy‐atorvastatin and *p*‐hydroxy‐atorvastatin was negligible, with both C_max_ and overall exposures remaining similar regardless of whether atorvastatin was given with or without asciminib.

A prior phase 1 study involving healthy participants analyzed the potential impact of asciminib 40 mg BID at steady state on the single‐dose PK of midazolam, a known substrate of CYP3A4. When midazolam was co‐administered with asciminib, there was a 28% increase in midazolam AUC_inf_, a 27% increase in the AUC_last_, and an increase of 11% in C_max_ relative to the PK parameters for midazolam administered alone.^24^ Based on physiologically‐based PK (PBPK) predictions at asciminib 200 mg BID, the interaction was somewhat higher, with an 88% and 58% increase in AUC_inf_ and C_max_ of midazolam, respectively.^8^ These observations suggest that asciminib is a weak inhibitor of CYP3A4 at all approved doses, and therefore, it is likely to be the contributing factor to the increased atorvastatin exposure in the current study.^24^

In vitro data have previously shown that asciminib could inhibit the transporters of atorvastatin (OATP1B1, OATP1B3, P‐gp, and BCRP).^9^ The value of CP‐1 as a biomarker that is indicative of OATP1B activity and OATP1B‐mediated DDIs has been previously highlighted.^27^ Based on in vivo findings, the selectivity of CP‐1 towards OATP1B has been established, making it a suitable biomarker for the evaluation of OATP1B activity.^28^, ^33^ In a review and analysis of CP‐1 data, significant correlations between CP‐1 AUC ratio (AUCR) or C_max_ ratio (C_max_R) and the AUCR of substrate drugs, including atorvastatin, in clinical studies were established. Specifically, CP‐1 C_max_R <1.25 was indicative of the lack of OATP1B‐mediated DDI (AUCR <1.25), while C_max_R <2 was associated with weak OATP1B‐mediated DDI (AUCR <2).^33^ A significant finding from the current study was that asciminib did not seem to affect the overall plasma CP‐1 concentrations. Also, AUC_0‐24_ G_mean_ ratio of atorvastatin plus asciminib versus atorvastatin alone was 1.02 (90% CI: 0.98‐1.05).

In another clinical DDI study, the effect of a single asciminib dose of 200 mg on CP‐1 was investigated. Based on the study results, no DDI effect on CP‐1 was observed at the 2.5‐fold higher dose of 200 mg (data on file). Overall, based on all available non‐clinical and clinical data, clinically relevant interactions between asciminib and OATP1B substrates are unlikely to occur at all approved asciminib doses, including 200 mg BID.

Our PBPK simulations indicated that the observed DDI effect on atorvastatin can be quantitatively explained by CYP3A4 inhibition, as a similar increase in atorvastatin exposure was simulated when the PBPK model included CYP3A4 inhibition as the sole perpetrator characteristic of asciminib (data on file). In consequence, the inhibition of P‐gp and BCRP by asciminib is not considered to be relevant in the current study of atorvastatin as a victim. This is consistent with the data showing that asciminib is a weak CYP3A4 inhibitor based on midazolam DDI,^24^ and the CP‐1 data generated in our study.

Both study treatments were well tolerated, and AEs were consistent with the known safety profile of asciminib.^34^, ^35^, ^36^, ^37^ No deaths or serious AEs were reported. The most reported AE by preferred term was pruritus, observed in 22.7% of participants, followed by increased heart rate and rash, each reported by 13.6% of participants. Notably, there were no AEs related to vital signs, clinical laboratory results, or ECGs, which suggests that the increases in atorvastatin exposure when given in combination with asciminib were well tolerated.

In conclusion, steady‐state asciminib 80 mg QD resulted in small increases in the exposure of atorvastatin, likely determined by CYP3A4 inhibition, and considered to be clinically irrelevant. CP‐1 concentrations remained unaffected by exposure to steady‐state asciminib, supported by additional data from another clinical study for a single asciminib dose of 200 mg, suggesting the lack of OATP1B inhibition at all approved doses. Multiple doses of asciminib 80 mg QD, given alone or concomitantly with atorvastatin 20 mg, were well tolerated by healthy participants. This study provides important results demonstrating the absence of DDI between asciminib and atorvastatin, and supporting the concomitant use of asciminib with atorvastatin.

## Author Contributions

*Conceptualization*: Matthias Hoch, Felix Huth, Michelle Quinlan, Shefali Kakar. *Data curation*: All authors. *Formal analysis*: Matthias Hoch, Michelle Quinlan, Seshulatha Jamalapuram, Amarnath Bandaru, Suleyman Eralp Bellibas, Asmae Mirkou. *Investigation*: All authors. *Methodology*: Matthias Hoch, Wendy Weis, Felix Huth, Seshulatha Jamalapuram, Michelle Quinlan, Amarnath Bandaru, Suleyman Eralp Bellibas. *Resources*: All authors. *Supervision*: All authors. *Validation*: All authors. *Writing – original draft*: All authors. *Writing – review & editing*: All authors.

## Conflicts of Interest

All authors are employees of Novartis. Matthias Hoch, Wendy Weis, Felix Huth, Michelle Quinlan, Seshulatha Jamalapuram, Suleyman Eralp Bellibas, Asmae Mirkou, Shruti Kapoor, and Shefali Kakar are shareholders of Novartis.

## Funding Information

This study was sponsored and funded by Novartis Pharma AG.

## Supporting information



Supporting Information

## Data Availability

Novartis will not provide access to patient‐level data, if there is a reasonable likelihood that individual patients could be re‐identified. Phase I studies, by their nature, present a high risk of patient re‐identification; therefore, patient individual results for phase I studies cannot be shared.
