# Supplementary material for: Assessment of Pharmacokinetic Drug Interaction of Asciminib with Atorvastatin in Healthy Participants
Source: Clin Pharmacol Drug Dev. 2025 Oct 22;15(2):e1611. doi: 10.1002/cpdd.1611 (PMC12856970; doi:10.1002/cpdd.1611)
Supplement: Supplementary file 1 — Supporting Information [file CPDD-15-0-s001.docx]

# Supplementary Online Material

**Assessment of pharmacokinetic drug interaction of asciminib with atorvastatin in healthy participants**

Matthias Hoch, PhD^1^, Wendy Weis, BSc^2^, Felix Huth, PhD^1^, Seshulatha Jamalapuram, PhD^2^, Michelle Quinlan, PhD^2^, Amarnath Bandaru, MD^3^, S. Eralp Bellibas, MD, PhD^2^, Asmae Mirkou, PharmD^4^, Shruti Kapoor, MBBS, MSc^2^, Shefali Kakar, PhD^2^

^1^Novartis Biomedical Research, Basel, Switzerland

^2^Novartis Pharmaceuticals, East Hanover, NJ, USA

^3^Novartis Healthcare Pvt Ltd, Rangareddy, Hyderabad, India

^4^Novartis Pharma AG, Basel, Switzerland

## **Figure S1.** Arithmetic mean (standard deviation) of asciminib trough plasma concentration-time profile (linear scale)


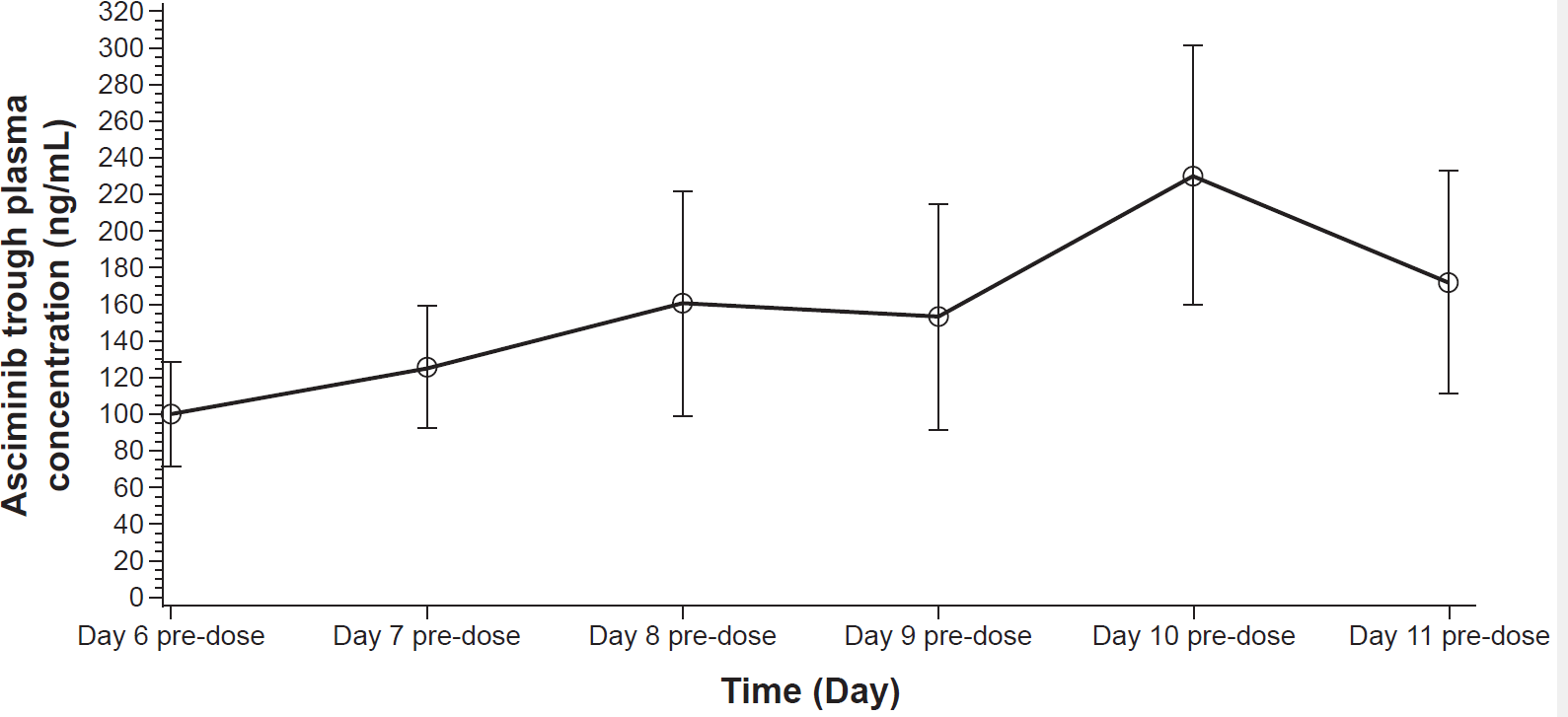


The first day of asciminib administration was on day 5, and the last day of asciminib administration was on day 11.
